# Supplementary material for: Antioxidant and Antidiabetic Activity of Cornus mas L. and Crataegus monogyna Fruit Extracts
Source: Molecules. 2024 Jul 30;29(15):3595. doi: 10.3390/molecules29153595 (PMC11314463; doi:10.3390/molecules29153595)
Supplement: Supplementary file 1 [file molecules-29-03595-s001.zip › molecules-3115339-Supplementary Material.pdf]

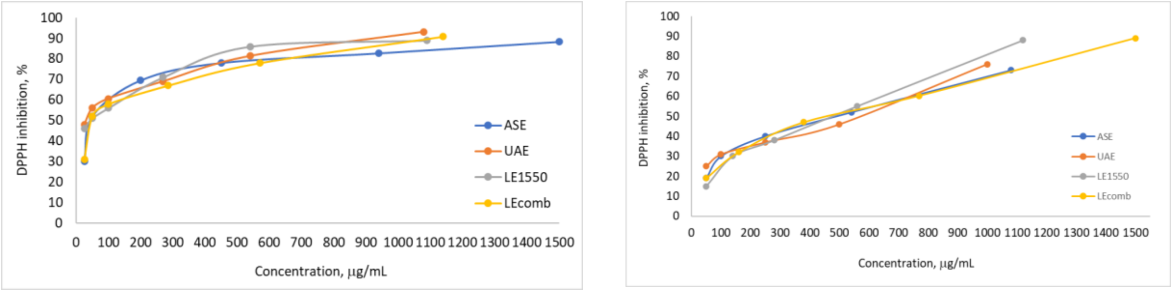

a b

Figure S1. Antioxidant activity of analyzed extracts of *C. mas* extracts (a) and *C. monogyna* extracts (b).

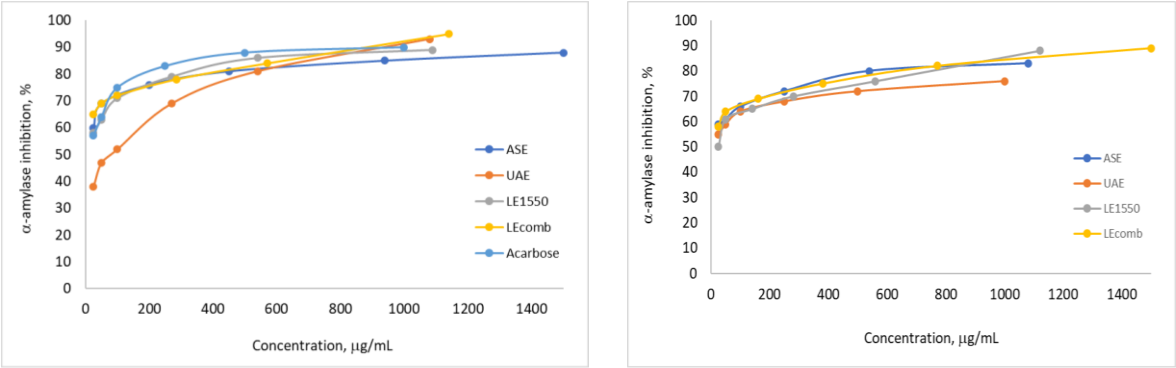

a b

Figure S2. α-amylase inhibition of *C. mas* extracts (a) and *C. monogyna* extracts (b).

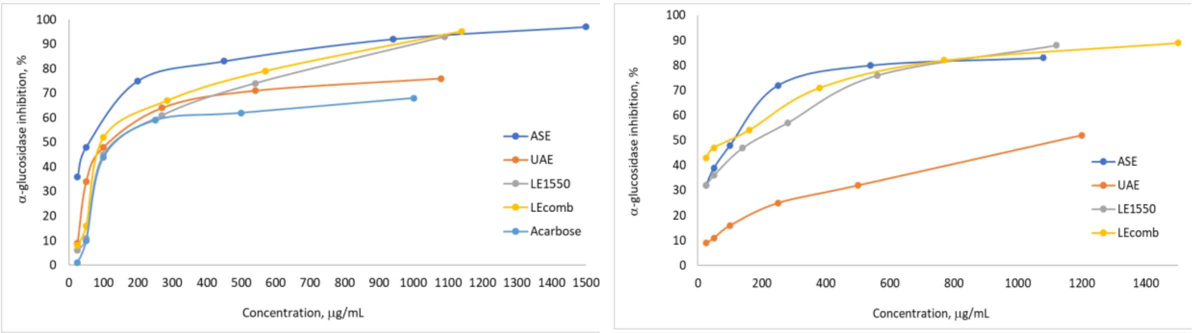

a b

Figure S3. α-glucosidase inhibition of *C. mas* extracts (a) and *C. monogyna* extracts (b).
